# Response of an Apex Mammalian Predator to an Emergence of 13-Year Periodical Cicadas

**DOI:** 10.3390/ani16030454

**Published:** 2026-02-01

**Authors:** Brian L. Cypher

**Affiliations:** 1 Cooperative Wildlife Research Laboratory, Southern Illinois University, Carbondale, IL 62901, USA; bcypher@esrp.csustan.edu; Tel.: +1-(661)-381-0048; 2 Endangered Species Recovery Program, California State University, Stanislaus, 1 University Circle, Turlock, CA 95382, USA

**Keywords:** *Canis latrans*, coyote, food habits, functional response, *Magicicada* spp., periodical cicada, resource pulse

## Abstract

Emergences of periodical cicadas constitute intense, short-duration resource pulses that can produce significant functional and numerical responses among insectivorous species that prey on them. The response of coyotes, apex predators in Illinois USA, to an emergence of 13-year periodical cicadas was investigated in 1989. Coyotes are not strongly insectivorous. However, during a 4-week period coinciding with peak emergence, cicadas were present in 85.9% of coyote scats (*n* = 71 scats) and 49% of scats contained only cicadas. Compared to the same time period in 1986–88 (*n* = 276 scats), use of all other food items was significantly lower in 1989 as was dietary diversity and overlap. This alteration in coyote foraging patterns constituted a strong functional response to the cicada emergence. The high abundance of cicadas combined with their ease of capture and consumption resulted in cicadas being an energetically efficient item for coyotes to forage upon. It is unknown whether other aspects of coyote life history (e.g., space use, activity patterns) also were affected or whether reduced predation pressure resulted in any effects on populations of their usual prey species.

## Simple Summary

During emergences of periodical cicadas, millions of the insects rapidly appear and constitute an abundant food resource for animals that consume insects. Coyotes are relatively large mammalian predators and insects generally are only a small part of their diet. However, during the 4-week peak of an emergence of 13-year periodical cicadas in Illinois USA, coyotes consumed primarily cicadas and substantially reduced consumption of their usual food items. Thus, even small prey can be important foods for large predators when the prey are very abundant and easily captured and consumed.

## 1. Introduction

A multitude of animal and plant species exhibit highly synchronized life history events, particularly ones associated with reproduction. Examples include masting by oaks (*Quercus* spp.) and other tree species [1], salmon (*Oncorhynchus* spp.) spawning runs [2,3], and mayfly (Order Ephemeroptera) emergences [4]. For the species involved, these events are characterized by a substantial increase in local abundance and, in part, this increase functions to overwhelm predators thereby facilitating sufficient reproductive success to ensure viable future populations of the species [5,6,7,8].

The increased abundance also constitutes a “resource pulse” for any predators that may feed on the species experiencing the increase [9]. These predators may themselves exhibit numerical or functional responses as a consequence of the resource pulse [1,8,9]. In southeastern New York USA, white-footed mouse (*Peromyscus leucopus*) and eastern chipmunk (*Tamias striatus*) abundance increased substantially in response to masting events by red oak (*Q. rubra*) [1]. In southwestern Virginia USA, white-footed mice and deer mice (*Peromyscus maniculatus*) expanded their reproductive period and their abundance increased concomitantly in response to masting events by red oak and white oak [10]. A vertebrate community of over 20 mammal and avian species increased in abundance, altered space use patterns, and formed larger groups in response to masting events by 21 Dipterocarpaceae in West Kalimantan, Indonesia [8]. Numbers of canopy gleaning magnolia warblers (*Dendroica magnolia*) and blackburnian warblers (*D. fusca*) increased significantly during an irruption of elm spanworm (*Ennomos subsignarius*), and other species altered their feeding strategies (e.g., greater canopy foraging) to exploit the caterpillars on two sites in Pennsylvania USA [11].

Periodical cicadas (*Magicicada* spp.) constitute an iconic example of a resource pulse [12]. These insects exhibit 13-year and 17-year life cycles in which individuals reside underground for almost their entire life feeding on the roots of deciduous trees [13]. In the spring of their 13th or 17th year, fifth (and final) instar nymphs emerge from the ground, molt into adults, and breed. Locally, millions of the insects emerge in just a few days with densities sometimes exceeding 3.7 million individuals per hectare (370/m^2^) with their biomass estimated at 230–428 kg/ha [14]. The adults breed and then die within a few weeks of emergence. Periodical cicadas are relatively large insects with bodies up to 33 mm long and 10 mm wide. The combination of large size, superabundance, and apparent lack of defenses such as toxins, stingers, or biting mouthparts [15,16,17] results in the emergences constituting extreme resource pulses that are exploited by numerous species of mammals, birds, reptiles, and arthropods [14,18]. Native peoples in eastern North American even apparently collected cicadas for food during emergences [18]. Periodical cicadas emerging in a given year and region are considered members of a specific brood designated by Roman Numerals; there currently are 12 broods of 17-year periodic cicadas and 3 broods of 13-year periodic cicadas extant. Each brood consists of one or more species [13].

Functional responses of vertebrates to cicada emergences have primarily been investigated among birds and small mammals [16,17,18,19,20,21]. With recent interest in the effects of resource pulses on ecosystems e.g., [1,9], including cicada emergences e.g., [12,13], I conducted a retrospective analysis of coyote (*Canis latrans*) food habit data collected during a periodical cicada emergence. In spring 1989, an emergence of Brood XXIII 13-year periodical cicadas occurred in southern Illinois USA where a multi-year (1986–1989) ecological study of coyotes was in progress [22]. This provided an opportunity to assess whether this relatively large bodied (ca. 9–14 kg) mammal that is an apex predator in the regional ecosystem would exploit this resource pulse, and if so, whether this might temporally affect its ecological patterns, particularly food item selection. The peak emergence occurred from approximately 15 May–15 June 1989. I compared coyote food item selection during this period to the same period during the 3 years prior to the emergence.

## 2. Materials and Methods

### 2.1. Study Area

I conducted an investigation of coyote foraging ecology from May 1986 to June 1989 on an approximately 300-km^2^ study area that encompassed Crab Orchard National Wildlife Refuge (CONWR) in Williamson and Jackson Counties, Illinois USA [22]. CONWR is located approximately 5 km east of the town of Carbondale and was established in 1947, primarily as a wintering area for Mississippi Flyway Canada geese (*Branta canadensis*). Topography at CONWR is characterized by gentle or flat terrain with soils comprised of mostly shallow silt loams originating from loess [23]. The climate is relatively warm and humid with mean January and July temperatures of 5 °C and 32 °C, respectively, and annual precipitation and snowfall averaging 124 cm and 28 cm, respectively [24].

The study area was within the western mesophytic forest region of the eastern deciduous forest biome [25]. Interspersion of vegetation communities was high due to past and present human disturbance (e.g., current farming and grazing and historic munitions production during World War II). Community types included upland forest, lowland forest, conifer plantations, old fields in various successional stages, agricultural fields, and wetlands [22]. Forested areas were primarily second growth. Upland forest associations were primarily oak-hickory (*Quercus-Carya*) and included red oak (*Q. rubra*), white oak (*Q. alba*), bitternut hickory (*C. cordiformis*), pignut hickory (*C. glabra*), sweetgum (*Liquidambar styraciflua*), black cherry (*Prunus serotina*), and flowering dogwood (*Cornus florida*) as common species. Lowland forests were dominated by cottonwood (*Populus deltoides*), sycamore (*Platanus occidentalis*), river birch (*Betula nigra*), American elm (*Ulmus americana*), and green ash (*Fraxinus pennsylvanica*). Plantations of loblolly pine (*Pinus taeda*) were distributed throughout the study area. Common understory species in forested areas included Japanese honeysuckle (*Lonicera japonica*) and poison ivy (*Toxicodendron radicans*). Old field habitats varied from primarily early-successional herbaceous associations dominated by fescue (*Festuca* spp.), foxtails (*Setaria* spp.), broomsedge (*Andropogon virginicus*), sweetclover (*Melilotus* spp.), goldenrod (*Solidago* spp.), or late boneset (*Eupatorium serotinum*) to late-successional shrub-sapling communities dominated by sassafras (*Sassafras albidum*), persimmon (*Diospyros virginiana*), multiflora rose (*Rosa multiflora*), blackberry (*Rubus* spp.), red cedar (*Juniperus virginiana*), and autumn olive (*Elaeagnus umbellata*).

Potential prey species for coyotes are CONWR [22] included white-tailed deer (*Odocoileus virginianus*), eastern cottontail rabbit (*Sylvilagus floridanus*), woodchuck (*Marmota monax*), muskrat (*Ondatra zibethica*), fox squirrel (*Sciurus niger*), prairie vole (*Microtus ochrogaster*), southern bog lemming (*Synaptomys cooperi*), white-footed mice, deer mice (*P. maniculatus*), northern bobwhite (*Colinus virginianus*), and various Passerifomes. Fruit-producing plant species included persimmon, black cherry, blackberry, American plum (*Prunus americana*), and pawpaw (*Asimina triloba*).

### 2.2. Food Item Use by Coyotes

To examine food item use by coyotes, scats (fecal) samples were collected biweekly along unpaved roads throughout the study area. Only scats exhibiting typical canid form and those at least 18 mm in diameter were collected to avoid inclusion of scats from smaller mammalian predators such as red foxes (*Vulpes vulpes*), gray foxes (*Urocyon cinereoargenteus*), and raccoons (*Procyon lotor*) [26,27]. Scats were placed in paper bags labeled with the collection date and location. Scats then were oven-dried in the bags at 60 °C for ≥24 h to neutralize any zoonotic parasite eggs and cysts. Contents of each scat were separated to identify food items. Mammalian remains were identified using macroscopic (e.g., length, texture, color, banding patterns) and microscopic (e.g., cuticular scale patterns) characteristics of guard hairs [28,29,30] and by comparing teeth and bones to reference guides [31,32] and specimens. Other vertebrates were identified to class and invertebrates to order, based on feathers, scales, and exoskeleton characteristics and comparison to reference specimens. Fruit seeds were identified at least to genus using a reference guide [33].

Food item use by coyotes was determined for the period 15 May–15 June 1989, which coincided with the cicada emergence in southern Illinois, and compared to this same period in 1986–1988. For statistical analysis, the data for 1986–1988 were combined and food items were grouped into six broader categories: deer, rabbits, medium sized mammals, small sized mammals, other items, and cicadas. Contingency table analyses and a chi-square test for independence were conducted to compare frequency of occurrence between 1989 and 1986–1988 sample sets for each food category. Yate’s correction-for-continuity was applied in all 2 × 2 contingency tables [34]. Shannon diversity indices (*H′*) were calculated for each of the two sample sets using the equation:

*H′* = (*N* log *N* − ∑*n_i_* log *n_i_*)/*N*

where *N* is the total number of occurrences of all items and *n_i_* is the number of occurrences of item *i* [35]. The diversity indices were compared using a Student’s *t*-test. Dietary overlap between the two sets was calculated using a niche overlap index [36]:

$$O_{kl}=\;\frac{\sum_{i}^{n}p_{il}p_{ik}}{\sqrt{\sum_{i}^{n}p_{il}^{2}\sum_{i}^{n}p_{ik}^{2}}}$$

where *O_kl_* is the resource overlap between sets *k* and *l*, and *p_i_* is the proportion of the diet comprised by item *i* for either set *k* or *l*. The range of values for *O_kl_* is 0–1 with values closer to 1 indicating high dietary overlap and values closer to 0 indicating low overlap. All statistical analyses were conducted using SPSS (SPSS Statistics package, ver. 29.0.1.1; IBM, Armonk, New York, NY, USA). An alpha level of 0.05 was used to determine statistical significance.

## 3. Results

### Food Item Use

The number of coyote scats collected and analyzed was 276 and 71 for the 1986–1988 and 1989 data sets, respectively. A diversity of food items was found in the scats (Table 1) including white-tailed deer, cottontail rabbit, woodchuck, muskrat, beaver (*Castor canadensis*), fox squirrel, raccoon, prairie vole, southern bog lemming, white-footed mouse, cow (*Bos Taurus*), Canada goose, eastern meadowlark (*Sturnella magna*), unidentified bird feather and eggshell, unidentified snake, unidentified reptile eggshell (probably turtle), June beetle (*Cotinis* spp.), grasshopper (Orthoptera), cicada, unidentified insect, American plum, blackberry, corn (*Zea mays*), and unidentified fruit flesh. The most frequently occurring items (>10%) were prairie voles, cottontail rabbits, adult deer, and fawns in 1986, cottontail rabbits, woodchucks, adult deer, and southern bog lemmings in 1987, cottontail rabbits, adult deer, woodchucks, and fawns in 1988, and cicadas and cottontail rabbits in 1989 (Table 1). The number of unique items found in scats each year was 17 in 1986, 18 in 1987, 20 in 1988, and just 10 in 1989.

**Figure 1:**
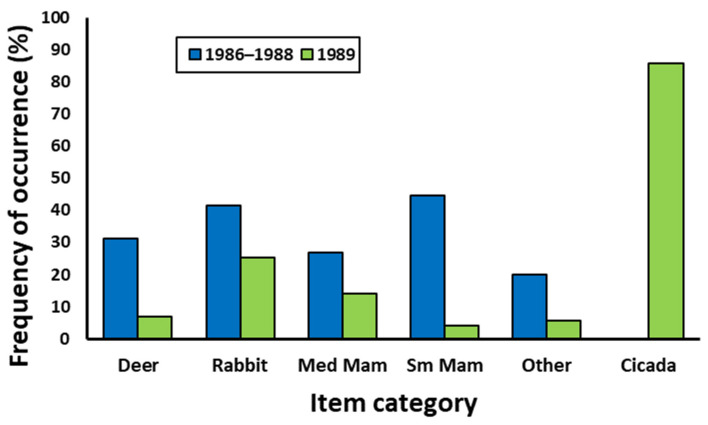
Frequency of occurrence of food items by category in coyote scats collected at the Crab Orchard National Wildlife Refuge in Illinois during the period 15 May–15 June in 1989 when an emergence of 13-year periodic cicadas occurred compared to the same period in 1986–1988 combined.

When food items were grouped into the broader categories, the most frequently occurring food items were small mammals in 1986 and 1987, rabbits in 1988, and cicadas in 1989 (Table 2). The Shannon diversity index ranged from 1.46 to 1.52 for 1986–1988 and declined to 1.24 in 1989. When data were combined for 1986–1988, the occurrence of food items in all categories was significantly higher for 1986–1988 compared to 1989 except for cicada which was significantly higher in 1989 (Table 2, Figure 1). The Shannon diversity index for 1986–1988 also was significantly higher compared to 1989 (*t*_105_ = 3.51, *p* < 0.001). Dietary overlap between 1986–1988 and 1989 was relatively low (*O_kl_* = 0.28).

## 4. Discussion

Coyotes are opportunistic omnivores that consume a diversity of food items [37,38]. This was evident on the CONWR study site where at least 22 different items were identified in coyote scats. Usually, insects appeared to be incidental items in coyote diets at CONWR as generally just one or two individual insects occasionally were found in scats comprised of other food items [22]. Most insects likely are consumed by coyotes when encountered opportunistically while foraging for other items. According to optimal foraging theory [39,40], animals should maximize foraging benefits (e.g., calories, essential nutrients) relative to foraging costs (e.g., searching, pursuing, capturing, consuming, detoxifying). Larger food items generally optimize foraging, but small items also can be consistent with optimal foraging if they are available in abundance and they are easily secured and consumed. A well-known example of this among terrestrial carnivores is the extensive summer-long consumption of army cutworm moths (*Euxoa auxiliaris*) by grizzly bears (*Ursus arctos horriblis*) [41,42].

**Figure 2:**
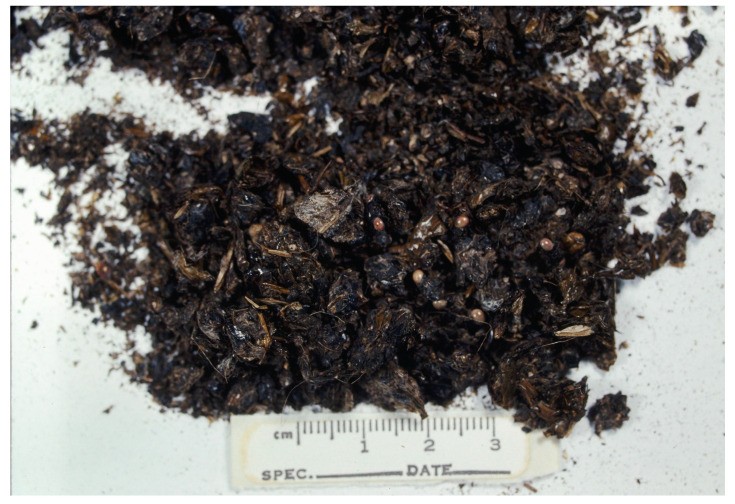
Coyote scat collected at Crab Orchard National Wildlife Refuge in Illinois in May 1989. The scat contents are exclusively periodical cicadas. The red eyes and wings of numerous cicadas are evident.

Emerging periodical cicadas clearly would constitute an abundant and easily secured resource that is high in protein and fat [15]. During the peak emergence period on the CONWR study site, I visually estimated a cicada density of ≥1/m^2^ at ground level. Thus, a foraging coyote easily could have rapidly secured and consumed a large number of cicadas and satisfied daily energy needs. Consequently, in spring 1989, cicadas quickly became the primary item in coyote diets. In addition to cicadas occurring in 85.9% of coyote scats during the 1989 sampling period, 49% of the scats contained only cicadas (Figure 2) and no other food items. This resulted in the lower number of items used and significantly lower item diversity in 1989 compared to previous years, and the low dietary overlap between 1989 and the other years.

Coyotes at CONWR also exploited other resource pulses including cripples and carcasses of white-tailed deer and Canada goose available during winter hunting seasons, and immense crops of wild persimmon fruits each fall [22]. These pulses involved much larger items and occurred annually whereas cicada emergences likely only occurred once during the life-time of most coyotes. Consumption of cicadas by coyotes has been reported previously [43,44,45,46], but the extent to which coyotes fed on cicadas in these other studies was not quantified.

Coyotes at CONWR exhibited a short-duration functional response to the periodical cicada emergence by significantly altering their foraging patterns. Other species, particularly primarily insectivorous ones, have exhibited a similar functional response by switching foods to feed primarily or even exclusively on cicadas during emergences [14,18]. In the mid-Atlantic region of eastern North America, 82 bird species were documented to shift foraging patterns during the 2021 Brood X emergence to exploit cicadas [19]. During an emergence in Kentucky USA in 1985, insectivorous short-tailed shrews (*Blarina brevicauda*), omnivorous white-footed mice, and even primarily herbivorous prairie voles (*Microtus ochrogaster*), included large quantities of cicadas in their diet (frequency of occurrence in stomachs was 93.8%, 95%, and 58.0%, respectively) [20]. During the 2004 Brood X emergence in Indiana USA cicadas were present in 66.7% and 77.3% of short-tailed shrew and raccoon (*Procyon lotor*) stomachs, respectively, and constituted over 51% of the volume of items [17]. Finally, in a situation analogous to the coyotes at CONWR, gray foxes and red foxes in east Tennessee USA shifted their diet and consumed primarily 17-year periodical cicadas during an emergence in spring 1987. During the peak emergence in May, cicadas were present in 100% of fox scats [47]. However, although they are canids like coyotes, gray and red foxes are considerably smaller, are not apex predators, and commonly are more insectivorous than coyotes [48].

Species also can exhibit other types of functional responses to cicada emergences in addition to altered food habits. The 2004 Brood X emergence extended into Ohio USA, and white-footed mice on one study site exhibited a higher density, number of reproductive females, and number of litters [48]. During the 2008 Brood XIV emergence in Massachusetts USA, two coyote family groups with young pups were observed to concentrate activity in areas of high cicada density [46]. Whether coyotes at CONWR exhibited other functional responses due to the abundance of cicadas, such as reduced activity or reduced space use, is unknown.

The responses of other species to periodical cicada emergences sometimes can alter, at least temporarily, ecosystem dynamics. In the case of the 2021 Brood X emergence, the shift by insectivorous birds to feeding on cicadas resulted in a significant reduction in predation on oak-eating caterpillars. Consequently, herbivory on white oak doubled and potentially reduced acorn production [19]. The altered food item use by coyotes at CONWR in 1989 may have been consequential for some prey populations. Use of white-tailed deer, rabbits, medium mammals, and small mammals was significantly lower in 1989 compared to the previous three years. Whether these species benefitted from this reduction in predation pressure is unknown. The reduction was only for the relatively short time of approximately four weeks during the peak cicada emergence. However, the emergence period also overlapped with the reproductive season of many of the prey species and conceivably reproductive success could have been higher for these species in 1989.

## 5. Conclusions

Emergences of periodical cicadas clearly can produce significant numerical and functional responses among insectivorous species. The results from the CONWR study site demonstrate that emergences also can have direct effects on species in higher trophic levels. In this case, the coyote, an apex predator that is not primarily insectivorous, exhibited a significant functional response by altering foraging patterns. Whether the consequential reduction in predation on other prey produced any sort of cascade effect on these species (e.g., increased reproductive success, increased abundance) is unknown. This likely could be easily determined through future investigation as the years in which emergences will occur and the regions where they will occur are predictable [13]. Thus, a priori studies could be designed and conducted to more precisely determine the effects of periodical cicada emergences on multiple trophic levels.

## Figures and Tables

**Table 1:** Frequency of occurrence of food items in coyote scats collected at the Crab Orchard National Wildlife Refuge in Illinois during the period 15 May–15 June in 1986, 1987, 1988, and 1989.

	Frequency of Occurrence (%)				
Food Items	1986 (*n* = 102)	1987 (*n* = 80)	1988 (*n* = 94)	1986–1988 (*n* = 276)	1989 (*n* = 71)
White-tailed deer—adult	23.5	11.3	18.1	18.1	1.4
White-tailed deer—fawn	16.7	7.5	13.8	13.0	7.0
Cottontail	34.3	32.5	57.4	41.7	25.4
Woodchuck	2.9	12.5	14.9	9.8	5.6
Muskrat	5.9	8.8	6.4	6.9	1.4
Beaver	0	1.3	0	0.4	0
Fox squirrel	4.9	1.3	4.3	3.6	4.2
Raccoon	5.9	2.5	9.6	6.2	2.8
Prairie vole	52.0	45.0	4.3	33.7	0
Southern bog lemming	9.8	11.3	0	6.9	0
White-footed mouse	0	0	1.1	0.4	0
Cow	1.0	0	1.1	0.7	0
Canada goose	3.9	3.8	1.1	2.9	1.4
Eastern meadowlark	2.0	0	0	0.7	0
Unknown bird	1.0	1.3	0	0.7	0
Bird eggshell	2.0	2.5	9.6	4.7	0
Unidentified snake	2.9	0	1.1	1.4	0
Reptile eggshell	2.9	1.3	5.3	3.3	0
June beetle	1.0	2.5	6.4	3.3	5.6
Grasshopper	0	2.5	0	0.7	0
Cicada	0	0	0	0	85.9
Unidentified insect	0	1.3	1.1	0.7	0
American plum	0	5.0	2.1	2.2	0
Blackberry	0	0	4.3	1.4	0
Corn	0	0	2.1	0.7	0
Unidentified fruit	0	0	1.1	0.4	0

**Table 2:** Frequency of occurrence of food items by category in coyote scats collected at the Crab Orchard National Wildlife Refuge in Illinois during the period 15 May–15 June in 1986, 1987, 1988, and 1989. Frequency of occurrence was statistically compared between 1986–1988 and 1989 for each category.

	Frequency of Occurrence (%)						
	1986	1987	1988	1986–1988	1989	1986–1988 vs. 1989	
Category	(*n* = 102)	(*n* = 80)	(*n* = 94)	(*n* = 276)	(*n* = 71)	*χ^2^*	*p*
White-tailed deer	40.2	18.8	31.9	31.2	7.0	15.75	<0.001
Rabbit	34.3	32.5	57.4	41.7	25.4	5.69	0.017
Medium mammal	19.6	26.3	35.1	26.8	14.1	4.32	0.038
Small mammal	61.8	56.3	5.3	44.6	4.2	38.01	<0.001
Other	16.7	20.0	34.0	19.9	5.6	7.19	0.004
Cicada	0	0	0	0	85.9	281.8	<0.001
*H’*	1.50	1.52	1.46	1.57	1.24		

## Data Availability

Data collected during this study are available upon reasonable request.
